# Engineering Climate-Change-Resilient Crops: New Tools and Approaches

**DOI:** 10.3390/ijms22157877

**Published:** 2021-07-23

**Authors:** Fahimeh Shahinnia, Néstor Carrillo, Mohammad-Reza Hajirezaei

**Affiliations:** 1 Bavarian State Research Centre for Agriculture, Institute for Crop Science and Plant Breeding, 85354 Freising, Germany; 2 Facultad de Ciencias Bioquímicas y Farmacéuticas, Instituto de Biología Molecular y Celular de Rosario (IBR-UNR/CONICET), Universidad Nacional de Rosario, Rosario 2000, Argentina; carrillo@ibr-conicet.gov.ar; 3 Department of Physiology and Cell Biology, Leibniz Institute of Plant Genetics and Crop Plant Research, 06466 Gatersleben, Germany

**Keywords:** cyanobacteria, photosynthesis, volatile compounds, gene expression, metabolites, plant protection resources, macro- and micronutrients

## Abstract

Environmental adversities, particularly drought and nutrient limitation, are among the major causes of crop losses worldwide. Due to the rapid increase of the world’s population, there is an urgent need to combine knowledge of plant science with innovative applications in agriculture to protect plant growth and thus enhance crop yield. In recent decades, engineering strategies have been successfully developed with the aim to improve growth and stress tolerance in plants. Most strategies applied so far have relied on transgenic approaches and/or chemical treatments. However, to cope with rapid climate change and the need to secure sustainable agriculture and biomass production, innovative approaches need to be developed to effectively meet these challenges and demands. In this review, we summarize recent and advanced strategies that involve the use of plant-related cyanobacterial proteins, macro- and micronutrient management, nutrient-coated nanoparticles, and phytopathogenic organisms, all of which offer promise as protective resources to shield plants from climate challenges and to boost stress tolerance in crops.

## 1. Introduction

Environmental stresses and nutrient limitation are among the major causes of crop losses worldwide, a trend that will likely worsen if current models of global warming prove correct [1]. Adverse environmental conditions for plant growth, e.g., poor soil conditions, nutrient deficiencies, drought, and pathogen attack, constitute the most relevant factors in agricultural yield reduction [2,3]. All these hardships cause oxidative stress in higher plants; oxidative stress is defined as a shift of the balance between pro-oxidative and antioxidative reactions [4], and results in the abnormal accumulation of excited and partially reduced forms of oxygen such as singlet oxygen (^1^O_2_), the superoxide radical (O_2_^•−^), and hydrogen peroxide (H_2_O_2_), collectively known as reactive oxygen species (ROS) [4,5]. Many adverse environmental conditions, including drought and salinity, are also accompanied by an associated osmotic stress resulting from a decrease in water availability [6,7,8].

When growing in their natural habitats, wild plants encounter multiple environmental constraints and have developed numerous strategies to survive and set seeds under unfavorable conditions. Selection of crop cultivars by breeding has been conducted with a bias towards high plant productivity within quite narrow environmental limits. During domestication, many traits mediating stress tolerance in wild ancestors were lost in modern bred cultivars. A comparison of the average yield of eight major crops indicates that the combination of biotic and abiotic stresses resulted in yield losses in the range of 60–90% (FAO, 2011, http://www.fao.org/docrep/014/mb060e/mb060e00.pdf, accessed on 21 July 2021) [2]. These observations indicate that there is a large potential for yield improvement and that engineering of stress tolerance could have a lasting impact in agricultural practice.

Traditional approaches to increasing plant stress tolerance have largely relied on the strengthening of endogenous protective mechanisms via the overexpression of the genes involved in their respective pathways and/or by limiting the key components of these systems. Endogenous responses are controlled by cascades of molecular networks involving stress perception, signal transduction, transcriptional regulation, and expression of specific stress-related effector genes [9]. Components of the signaling and transcriptional regulation pathways have been extensively manipulated. These include members of the dehydration-responsive element-binding (DREB) protein family, transcription factors (TF) involved in abscisic acid (ABA)-dependent pathways, and others belonging to the Nuclear Factor Y family and the ABA-independent AP2/ERF and NAC families [10,11,12]. Most attempts, however, have been directed towards the overexpression of effector genes acting downstream of the plant response cascade, including ion channels [13], enzymes involved in the synthesis of compatible osmolytes [14,15], ROS scavengers, and other antioxidant proteins [16].

Although increased stress tolerance has generally been achieved through these approaches under controlled conditions, translation to the field has met with considerable difficulties, in part due to the complexity of the endogenous regulatory networks governing these responses. Overexpression of genes that operate upstream of the response cascade (e.g., signaling factors or transcriptional regulators) often leads to growth and/or reproductive penalties, whereas boosting the levels of functional effector proteins confers tolerance to a limited set of stresses or even a single source of stress, which is of relative significance to field conditions, where the combination of concurrent stresses (for instance, heat plus drought) is the rule rather than the exception [9].

These limitations have prompted the search for novel, alternative approaches to improve crop productivity in suboptimal environments, and a number of new strategies are being explored to extend and diversify the toolkit of biotechnological resources. Within this context, manipulation of phytohormone levels has emerged as a most promising choice, since most of these signaling molecules have been shown to participate in plant responses to adverse environmental conditions—most conspicuously ABA, the canonical stress hormone. In addition, Xu et al. [17] performed a transcriptome analysis in creeping bentgrass under drought stress and showed that hormone signaling and synthesis, particularly synthesis of cytokinins, play a crucial role. The relationships between phytohormones and secondary metabolites and their effects on drought tolerance in crop plants have been recently reviewed as indicating that they significantly contribute to better plant development under drought in crops [18]. Engineering of cell wall metabolism has also attracted considerable attention as a possible means to increase tolerance to multiple stresses via a physical barrier against abiotic and biotic onslaughts [19,20]. Exploiting plant interactions with beneficial microorganisms provides still another novel approach for improving growth and yield in adverse environments. Successful interventions based on these various strategies have been comprehensively addressed in a series of excellent reviews [18,21,22,23], and will therefore not be discussed here in great detail.

A critical and, until recently, relatively unexplored aspect of the survival and reproduction of plants exposed to environmental hardships is nutrient status. Specifically, improvements of the activity of energy and sugar metabolic pathways have been shown to correlate with increased plant development and tolerance to drought and other stresses [24,25]. Chloroplasts play a key role in this context, as the site of carbon, nitrogen, and sulfur assimilation and of phytohormone synthesis. At the same time, they are also primary targets of environmental hardships and oxidative stress. Preservation of chloroplast metabolic routes, especially photosynthesis, is thus of paramount importance for the survival and growth of stressed plants. While increasing the stock of antioxidant systems, as described before [5], provides protection against individual ROS, the use of alternative electron sinks has gained momentum in recent years as a way to prevent generation of all chloroplast oxidants and develop multiple stress tolerance [5].

A direct approach to improving nutrient availability is via exogenous supplementation, which is probably as old as agriculture itself. New findings, however, have revealed previously unknown links with stress tolerance and provided novel tools for both customized nutrient delivery and better assimilation via the manipulation of biochemical and morphological traits associated with nutrient uptake and mobilization. Finally, several studies have shown that plant–microbe interactions can influence abiotic stress tolerance, with significant impacts on growth and biomass accumulation. While the role played by endophytic microorganisms has been known for some time and is associated with their modulation of phytohormones production in the plant host [22,23], further discoveries have shown that beneficial effects are not restricted to rhizosphere microorganisms but also include phytopathogens, which act via entirely different mechanisms that mainly occur in the chloroplasts [26].

The present review is thus focused on a critical assessment of these novel approaches, the molecular mechanisms underlying their protective effects, and their application towards increased stress tolerance of model and crop plants. Emphasis is placed on metabolic responses and nutritional status, with several examples illustrating the correlation between preservation of metabolism and stress tolerance. The knowledge gained through these studies may serve as a valuable resource for the customized design of crops with higher biomass production in a sustainable manner in marginal lands and under suboptimal environmental conditions.

## 2. Introduction of Alternative Electron Sinks Protects Chloroplast Metabolic Activities under Stress

### 2.1. Chloroplasts as Targets and Sensors of Environmental Stresses

Photosynthesis is very sensitive to environmental stresses, and photosynthetic capacity generally declines before other cellular functions [27,28]. At the same time, chloroplasts play an important role in plant adaptation to adverse situations [29], as they are the centers of key metabolic processes and act as environmental sensors to perceive stress. Carbon assimilation via the Calvin–Benson cycle (CBC) is particularly sensitive to drought and other environmental challenges, even under conditions in which photosynthetic electron transport remains functional [30]. Starch, the transitory storage carbon, is considered a crucial molecule in plants exposed to abiotic stresses [31]. The degradation of starch during photosynthesis-limiting conditions provides carbon and energy to sustain plant survival, and the released soluble sugars act as osmoprotectants that stabilize cell turgor and membrane integrity [32]. In drought-stressed plants, nitrogen metabolism is downregulated [33] while the levels of certain amino acids are enhanced, since they can be beneficial for stress acclimation. For instance, proline acts as an osmolyte and is also involved in ROS scavenging [34], along with the nonproteinogenic amino levulinic acid [35,36]. The physiological and molecular responses of plants under stress conditions are also associated with the energy status of the cells [37,38]. In the chloroplasts, ATP and NADPH are used to drive several metabolic reactions, transport pathways, and repair mechanisms, such as turnover of the D1 protein from photosystem (PS) II, which help plants to cope with the detrimental consequences of the stress condition.

Inactivation of the CBC leads to a decline in the content of the terminal electron acceptor NADP^+^, which in turn causes over-reduction of the photosynthetic electron transport chain (PETC) [39]. Under such conditions, the excess of energy and reducing equivalents at the PETC can be delivered to O_2_ to generate ROS, with the consequent threat of oxidative damage to proteins, membranes, and photosystems. These reactive species can be synthesized in various cellular compartments including plastids, mitochondria, peroxisomes, the cytosol, and the apoplast; as byproducts of oxidoreductive pathways (photosynthesis, respiration, photorespiration); or by committed enzymes such as apoplastic NADPH oxidase [5]. Chloroplasts, and to a lesser extent peroxisomes, are the main sources of ROS in the photosynthetic tissues of stressed plants [5]. Under short-term stress conditions, chloroplasts activate photoprotective mechanisms including alternative electron transport (AET) pathways, photorespiration, and ROS-scavenging enzymes. Under prolonged stress, the ratio of PS II to PSI, the size of the antenna, and the levels of chlorophyll pigments are reduced. Plants also show a decrease in most PS proteins, specifically Lhcb5, Lhcb6, and PsbQ [34]. Carotenoids, which act as light-harvesting pigments, can also function as antioxidants that protect PSI/II from photodamage under stress conditions by scavenging ROS [40]. In addition, plant stress responses usually involve increased synthesis of antioxidant metabolites such as glutathione and ascorbate [10], as well as activation of AET pathways and dissipative systems that minimize misrouting of energy and reducing power to oxygen. All these protective systems offer opportunities for genetic intervention. It is worth noting that scavenging systems, either enzymatic or nonenzymatic, combat oxidative stress after ROS have been generated; preventing ROS formation in the electron transport chains of chloroplasts or mitochondria is expected to be even more effective [5].

A major photoprotective mechanism that allows plants to dissipate excess light energy absorbed in the light-harvesting complex of PSII is based on the release of the extra energy in the form of heat through nonphotochemical quenching (NPQ). Activation of NPQ in photosynthetic organisms is triggered by an increase in lumenal pH, which in turn depends on the activity of two different cyclic electron transport (CET) pathways around PSI [41,42,43,44]. Several other AET pathways and redox valves are functional in chloroplasts, underscoring the relevance of these dissipative systems for stress protection. They include the NADP^+^-dependent malate dehydrogenase (NADP–MDH) valve, which exports NADPH from the stroma to the cytosol through the malate–oxalate shuttle, photorespiration in C3 plants, and chlororespiration mediated by the chloroplast alternative oxidase [45,46]. Finally, genes associated with ROS scavenging are induced under stress conditions, as their products detoxify ROS and prevent oxidative damage.

As indicated before, engineering strategies aimed at improving stress tolerance have mostly focused on the overexpression of plant endogenous genes belonging to molecular networks for stress perception, antioxidants, or dissipative pathways [5,47,48,49]. A variant of this general approach is the introgression of alleles present in wild, stress-tolerant relatives of present-day crops via either traditional breeding or genetic engineering. Description of the results obtained in this very active field of research is beyond the scope of this article, but comprehensive accounts of these advances can be found in various recent reviews (see, for instance, [50]). Again, the success of these strategies has been limited by the existence of regulatory systems in the host to which the endogenous genes belong. They might restrict the possibilities of overexpression and generate pleiotropic effects, although the use of stress-inducible and/or synthetic promoters provides a way to overcome this limitation [51].

A new approach that has been recently applied and has shown considerable promise is based on the introduction of genes encoding electron carrier proteins that are not found in plants (at least in angiosperms) but are functional in photosynthetic microorganisms. These genes are generally not recognized by endogenous regulatory systems (see, however, [52]), so their expression can be customized to act as electron sinks of the PETC, provided that they still interact in a functional way with their plant counterparts despite their evolutionary distance, and to improve stress tolerance, growth, and yield of the transgenic host. Successful examples of this approach are discussed in some detail in the following sections.

### 2.2. Cyanobacterial Flavodoxin as a Tool to Achieve Plant Tolerance to a Broad Range of Environmental Adversities

Cyanobacteria can survive under fluctuating aerobic and anaerobic conditions, and thus provide a rich source of genes for plant genetic engineering due to their having both similarities with and differences from plant genetic systems. While these photosynthetic prokaryotes share many metabolic pathways with plant cells, and especially with chloroplasts, most mechanisms involved in plant regulation of gene expression have arisen after endosymbiosis, leaving ample room for customized manipulation of introduced traits. Among the cyanobacterial proteins involved in tolerance to environmental and/or nutrient stresses in these microorganisms, the electron transfer shuttle flavodoxin (Fld) constitutes a paradigmatic case. Fld contains flavin mononucleotide as prosthetic group and is isofunctional with ferredoxin (Fd), an iron–sulfur protein present in all photosynthetic organisms ranging from cyanobacteria to plants. Fd is the final electron acceptor of the PETC and is essential for the distribution of low-potential reducing equivalents to energy-demanding metabolic steps in CO_2_ fixation, nitrogen, and sulfur assimilation; amino acid synthesis; and fatty acid desaturation, as well as many regulatory (e.g., thioredoxin (Trx) redox regulation system) and dissipative pathways [9]. Fd levels experience a considerable decrease in response to environmental stresses and other sources of ROS production, which decrease not only the expression of Fd but also its activity [53]. Similarly, iron deficiency also leads to diminished Fd levels [54], whereas its abundance increased with increasing iron availability in marine phototrophs [55].

Photosynthetic microorganisms like cyanobacteria and certain algae induce expression of Fld under both environmental stresses and iron starvation, as an adaptive response to compensate for the negative consequences of Fd decrease [53,54]. Fd substitution results in the restoration of electron delivery to energy-producing pathways such as NADP^+^ photoreduction and CO_2_ assimilation, thereby preventing misrouting of reducing equivalents to O_2_ and concomitant ROS production. The net outcome is an augmented tolerance towards various conditions of stress in algae and cyanobacteria.

Fd is regarded as one of the oldest protein signatures on Earth, having appearing long before the advent of oxygenic photosynthesis and the great oxidation event [56]. The Fld protein fold is also very ancient, barely younger than Fd, suggesting that this substitutive mechanism already had selective value in the anaerobic, highly reducing environment of the Archean world [56]. The presence of Fld in algae indicates that both Fd and Fld were present in the endosymbiont that gave origin to all lineages of photosynthetic eukaryotes [54]. The disappearance of Fld from the genomes of *Viridiplantae* has been associated with the transition of the green algal lineage from the open ocean to coastal waters (and from there to land), correlating with changes in iron bioavailability across habitats: low in the ocean, high on the coast [54,57].

**Figure 1:**
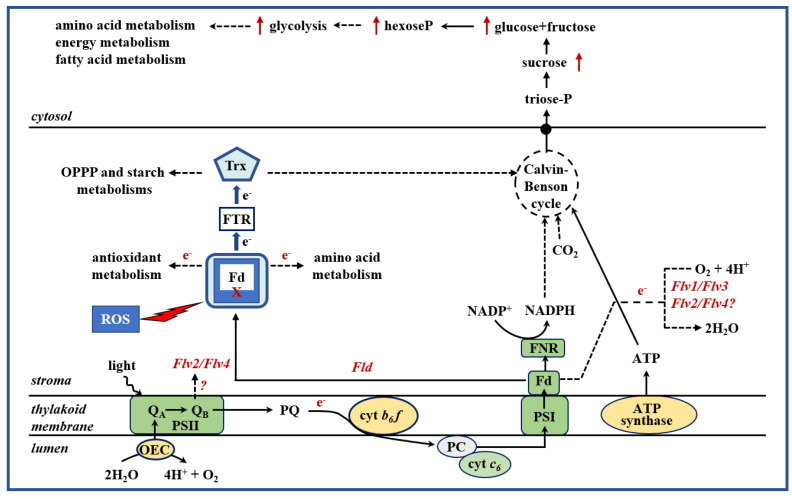
Schematic representation of the AET pathways associated with alternative electron carriers expressed in plants. Inactivation of the photosynthetic machinery caused by environmental constraints such as drought results in an overproduction of reactive oxygen species (ROS) and a decline of ferredoxin (Fd), the final acceptor of the photosynthetic electron transport chain, which in turn negatively affects the reduction of thioredoxin (Trx) by Fd-Trx reductase (FTR). This system participates in the redox modulation of components of the Calvin–Benson cycle, starch biosynthesis, the oxidative pentose phosphate pathway (OPPP), and other metabolic routes. Flavodoxin (Fld) compensates for the loss of stress-sensitive Fd and maintains electron flow to productive routes of the chloroplast, resulting in improved metabolic activity and plant development. In contrast to Fld, flavodi-iron (Flv) proteins act as electron sinks by capturing the excess of electrons and transferring them to oxygen to produce water without the formation of ROS. Cytochrome *c*_6_ (cyt *c_6_*) participates in the intersystem electron shuttle. Improved electron flow through photosynthesis and other redox pathways enables plants to maintain the energy balance via a higher provision of redox equivalents and to improve their overall metabolic response, allowing them to survive multiple stress conditions that exert oxidative damage. cyt *b*_6_*f*: cytochrome *b*_6_*f*; FNR: Fd-NADP^+^ reductase; OEC: oxygen-evolving complex; PC: plastocyanin; PQ: plastoquinone; PS: photosystem.

Introduction of a plastid-directed cyanobacterial Fld in tobacco proved that the flavoprotein was still able to engage with chloroplast redox partners in electron transfer reactions (Figure 1) [9,58]. The transgenic lines displayed a remarkably increased tolerance to multiple sources of environmental stresses, both abiotic [58] and biotic [59,60], as indicated by significant improvements in survival, growth, photosynthesis, and metabolic activity. They were also able to thrive in iron-limited media that were deleterious to their wild-type (WT) siblings [61]. Stress-dependent ROS accumulation was largely prevented in the transformants [58]. In vitro assays showed that prokaryotic Fld can act as an electron donor to several plant Fd-dependent enzymes including Fd-NADP^+^ reductase and Fd-Trx reductase [58]. Moreover, it preserved the activation state of key plastidic enzymes that depend on the Fd-Trx system, such as phosphoribulokinase and fructose 1,6-bisphosphatase [58]. As a result, the levels of many central metabolites belonging to the CBC, energy storage, and anabolic routes, as well as the contents of most amino acids, were significantly higher in the transformants [59,61]. Fld effects were dose-dependent, as expected for an electron carrier, and chloroplast location was mandatory [52,58].

Functional replacement of declining Fd was responsible for the protective effects, as confirmed by successful in vivo complementation of Fd-deficient tobacco plants [62]. It is noteworthy that incorporation of Fld into the oxido-reductive network of chloroplasts affected the chloroplast redox poise and led to phenotypic effects even in the absence of stress [63]. Improved stress tolerance resulting from expression of a plastid-targeted Fld has been also demonstrated in other angiosperms, including barrel clover [64], creeping bentgrass [65], and potato [66].

Taken together, these results show that Fld expression compensated for Fd decline during environmental hardships and iron deficiency by successfully engaging in at least some of the Fd-dependent pathways of plant chloroplasts. The resulting transgenic plants were able to withstand a remarkable range of stresses, including high temperature, chilling, drought, UV radiation, pathogens, and exposure to redox-cycling herbicides, all of which generate ROS in chloroplasts as a common feature [9,58,64,65,66,67].

### 2.3. Expression of Plastid-Targeted Algal Cytochrome c_6_ in Plants Increases Growth and Biomass Accumulation

In plant chloroplasts, reducing equivalents are shuttled between the cytochrome *b*_6_*f* complex and PSI by plastocyanin (PC), a copper-containing luminal protein, but some algae and cyanobacteria also express the isofunctional electron carrier cytochrome *c*_6_ (cyt *c*_6_), which contains heme as prosthetic group [68]. Since phototrophic bacteria performing anoxygenic photosynthesis use cytochromes rather than PC as soluble electron shuttles, it is generally accepted that cyt *c*_6_ was the ancestral carrier in the primitive cyanobacteria, with the subsequent incorporation of PC providing a selective advantage for survival under iron deficiency [69]. As in the case of Fd and Fld, the original endosymbiont expressed the two proteins, but most members of the red algal lineage retained only cyt *c*_6_ [70]. Some species within the diatoms, which are products of a secondary endosymbiotic event, have gained a PC in more recent times by horizontal gene transfer from green algae [69]. The cyt *c*_6_ variants from the two lineages diverged significantly, with those present in red algae showing improved turnover efficiency at the expense of binding affinity [69]. It is assumed that loss of cyt *c*_6_ in plants reflects the dynamics of metal availability in terrestrial habitats, but no further evidence is available to substantiate this notion.

Lumen-targeted cyt *c*_6_ proteins from both algal lineages have been expressed in model angiosperms (Figure 1). Chida et al. [71] introduced the cytochrome from the red alga *Porphyra yezoensis* in Arabidopsis chloroplasts, whereas Yadav et al. [72] transformed tobacco plants with the cyt *c*_6_ gene from *Ulva fasciata*, a green alga. Despite the functional differences between the two types of carriers, incorporation of any of them into the chloroplast PETC resulted in transgenic plants exhibiting improved growth, increased leaf and root biomass, and higher contents of chlorophyll, protein, and/or starch [71,72]. Similar results were obtained in field-grown tobacco plants expressing cyt *c*_6_ from *Porphyra umbilicalis* [73]. Growth improvements were associated with increased photosynthetic rates and water use efficiency in the cyt *c*_6_ transformants [71,72,73]. Their stress tolerance was not assayed.

### 2.4. Flavodi-Iron Proteins Act as Electron Sinks and Enhance Plant Growth under Drought Stress Conditions

Another unexploited source of biotechnological potential lies in flavodi-iron proteins (FDPs), a subclass of redox-active proteins widely distributed among bacteria (including cyanobacteria), archaea, and eukaryotic phototrophs [74]. They can catalyze the reduction of O_2_ directly to water and of nitric oxide (NO) to nitrous oxide, thus protecting bacteria against ROS and NO [75]. All FDPs contain two redox centers, a flavin mononucleotide that functions as an electron acceptor and a non-heme Fe–Fe center that serves as active site [75]. Unlike bacterial FDPs, those present in photosynthetic organisms possess an additional NAD(P)H-flavin oxidoreductase module fused at the C-terminus [74]. Plants and algae contain two FDP isoforms, and cyanobacteria up to six. They are reported to act as heterodimers (named Flv1/Flv3 and Flv2/Flv4 in *Synechocystis*), although many aspects of their function at the molecular level remain to be elucidated. The Flv1/Flv3 complex is distributed in all photosynthetic organisms with the single notorious exception of angiosperms [74]. Flv2 and Flv4, in contrast, are only found in β-cyanobacteria [76]. They can relieve the excess of excitation pressure on the PETC by acting as electron sinks at various sites of the chain. Studies in *Synechocystis* cells have shown that the Flv1/Flv3 complex is reduced by PSI [77], whereas Flv2/Flv4 can engage in electron transfer with the primary acceptor Q_B_ of PSII [78,79]. Further research, however, revealed that Flv2/Flv4 can also operate as an electron sink at PSI, although the specific electron acceptor of this reaction was not identified [80]. The two complexes are associated with cyanobacterial stress responses, especially fluctuating light (Flv1/Flv3) and high irradiation (Flv2/Flv4). Loss of these adaptive resources from angiosperms has been attributed to a major increase in the efficiency of photorespiration [81] and CET [82] in flowering plants. Indeed, Wada et al. [83] showed that Flv1/Flv3 expression had no phenotypic effects on WT rice plants but significantly improved the photosynthetic efficiency of mutant lines deficient in CET.

Since FDPs are not existent in angiosperms, the question remains whether Flv1–4 can assemble therein as functional complexes and display the same biochemical activity as in lower phototrophs in order to improve stress tolerance. We tested this possibility by expressing *Synechocystis* Flv2/Flv4 in chloroplasts of tobacco and Arabidopsis (Figure 1). Flv-expressing plants exhibited increased tolerance toward various stresses including drought, as reflected by better growth and preservation of photosynthetic activity and membrane integrity [84]. Metabolic profiling under drought showed enhanced accumulation of soluble sugars and amino acids in transgenic Arabidopsis and a remarkable shift of sucrose into starch, in line with the metabolic responses of drought-tolerant genotypes [84]. The results indicate that the Flv2/Flv4 complex retained its stress-protective activities when expressed in chloroplasts of angiosperms by acting as an additional electron sink through functional interaction with the chloroplast PETC, despite the evolutionary divergence between cyanobacteria and angiosperms.

To evaluate the contribution of Flv1/Flv3 to the growth of plants exposed to drought in crops, the *Synechocystis* genes encoding these two proteins were expressed in barley, with their products being targeted to chloroplasts (Figure 1). The heterologous expression of Flv1/Flv3 accelerated days to heading, increased biomass, promoted the number of spikes and grains per plant, and improved the total grain weight per plant of transgenic lines exposed to drought [85]. Better growth was correlated with enhanced availability of soluble sugars, a higher turnover of amino acids and the accumulation of lower levels of proline in the leaf. Flv1/Flv3 maintained the energy status of the leaves in the stressed plants by converting sucrose to glucose and fructose, immediate precursors for energy production to support plant growth under drought [85]. The results suggest that sugars and amino acids play a fundamental role in the maintenance of energy status and metabolic activity to ensure growth and survival of plants exposed to drought (Figure 1). Engineering chloroplasts by introducing FDP-encoding genes into the plant genome, therefore, has the potential to constitute a novel biotechnological tool to generate plants with increased tolerance and metabolic performance under agronomically relevant stresses, and to improve plant productivity wherever drought stress represents a significant production constraint.

## 3. Increasing Accessibility and Mobilization of Macro- and Micronutrients to Improve Plant Stress Tolerance

A critical effect of drought on plant development is the limitation of access to macro- and micronutrients, which are taken up together with water by the roots and transported to the vegetative organs. The shortage of water results in severe structural and biochemical changes in plants, and nutrient management is one of the promising strategies to avoid their deficiency under drought conditions [86]. This can be accomplished by direct nutrient supplementation and/or by engineering the architecture of the plant, and especially the root, which plays a crucial role that defines the access to available water and soil nutrients.

Exogenous supplementation of nutrients, especially in field-grown plants, may be a feasible tool to circumvent harsh conditions, including drought. While this is a very ancient resource in agriculture, recent developments have revolutionized this traditional practice and revealed unsuspected links to stress tolerance. In this respect, two main approaches have been used to enhance plant growth under stress: foliar application of specific nutrients and delivery of various nanocoated fertilizers.

Ul-Allah et al. [87] showed in field experiments that the application of potassium on diverse hybrid maize plants under water-limited conditions significantly alleviated the drought susceptibility of all lines and improved yield traits. Furthermore, foliar application of zinc in field trials on wheat plants at both vegetative and reproductive developmental stages resulted in increased chlorophyll contents, water use efficiency, and grain yield, demonstrating that a short-term application of nutrients might be a suitable tool with which to improve productivity and grain nutrient content under water shortage [88].

Another promising strategy that has been developed in recent years by various scientists is the use of nanocoated macro- and/or micronutrients. In this respect, the effect of three micronutrient nanoparticles, zinc oxide (ZnO), boron trioxide (B_2_O_3_), and copper oxide (CuO), have been evaluated through foliar or soil application to soybean plants under drought conditions. Dimkpa et al. [89] reported that nanocoated nutrients were more effective when applied to the soil compared to foliar application. The treatments resulted in a reduction of drought symptoms by increasing the availability of several nutrients including nitrogen, potassium, zinc, boron, and copper, which improved shoot growth and final grain yield [89]. In a follow-up study, Dimkpa et al. [90] showed that the application of coated urea, a nitrogen-containing substance, with low amount of ZnO in two different locations and under drought stress conditions resulted in a better performance of wheat plants and an enhanced uptake of zinc, emphasizing the potential of this technology for field experiments.

The beneficial effects of foliar application of ZnO nanoparticles were also shown in field trials conducted in two consecutive years with eggplants exposed to drought stress conditions [91]. The authors reported that the acquisition of nutrients was improved, followed by an enhanced photosynthetic activity that finally resulted in a better growth performance of eggplants in a dry land region. Moreover, Astaneh et al. [92] demonstrated that nanofertilizers might be a beneficial tool to manage the loss of specific nutrients under abiotic stress conditions. They evaluated the effect of nanochelated nitrogen and urea fertilizers on the physiological characteristics of wheat plants under drought in two different field locations and demonstrated that the applied nanofertilizers improved nutrient remobilization, photosynthetic activity, and protein and nutrient contents, including phosphorous and potassium. Furthermore, Ahmadian et al. [93] showed recently that the application of nanozinc and nanosilica fertilizers to field-grown wheat plants improved the growth characteristics, including grain protein content, water content, cell membrane stability, and final grain yield, in two different experiments performed in two successive years.

Attempts to improve nutrient uptake via genetic manipulation of plant morphology have also shown considerable potential. For instance, Kato et al. [94] reported that the calcium-binding protein PCaP2, located in the plasma membrane, is involved in root hair development and acts as a signal transducer in Arabidopsis. During this study, the overexpression of the 23-residue N-terminal domain of PCaP2 (N23) employing the root-hair-specific EXPANSIN A7 promoter resulted in the suppression of root hair emergence and elongation, suggesting that the N23 domain of PCaP2 negatively regulates root hair tip growth via processing of calcium and phosphatidylinositol phosphate signals on the plasma membrane [94]. Tanaka et al. [95] used these same lines to investigate the physiological significance of root hairs, and found that N23-expressing plants did not produce root hairs even under phosphate-deficient conditions or after ethylene treatment, which typically stimulates their production. They also observed that these plants displayed a 47% reduction in water absorption, decreased drought tolerance, and a lower ability to adapt to heat compared to the wild type, emphasizing that root hairs play an important role in the absorption of water and thus in the availability of nutrients for better growth of plants under drought stress.

Furthermore, Fenta et al. [96] demonstrated in field experiments with three soybean cultivars (a sensitive cultivar with a shallow root phenotype, an intermediate cultivar with an intermediate root phenotype, and a tolerant cultivar with a deep-rooting phenotype) that a positive correlation could be observed between nodule size, above-ground biomass, and seed yield under well-watered and drought conditions. The authors concluded that the deep-rooting cultivar performed best under drought and that root phenotypic markers have great potential for use in screening soybean cultivars for drought tolerance under field conditions.

Recently, Ramireddy et al. [97] generated transgenic barley plants with an enlarged root system through the stimulation of cytokinin breakdown in roots via the expression of a cytokinin oxidase/dehydrogenase under the control of root-specific *EXPRESSED PROTEIN* (*EPP*) and *PEROXIDASE PROTEIN* (*PER*) promoters of rice, in order to explore the potential of cytokinin modulation in improving root functions. They demonstrated that the modification of root growth and branching via cytokinins, as negative regulators of root growth, resulted in an increase of macro- and microelement levels in the leaves and seeds of transgenic barley plants. This was accompanied by an enhanced tolerance to long-term drought, emphasizing that root engineering of cereals is an attractive tool to circumvent nutrient deficiency in agronomically important plants.

In addition to the severe effects of drought on roots, it can also initiate the senescence program in leaves, which is closely associated with the conversion of storage assimilates such as starch into soluble sugars and/or nutrient remobilization from senescing leaves. The released sugars and nutrients are translocated to sink organs such as roots, seeds, or sink leaves, and thus compensate for the loss of nutrients caused by water shortage. The function of starch biosynthesis and of the soluble carbohydrates on sugar homeostasis and transport in drought conditions were recently described in detail by AbdElgawad et al. [98] and Saddhe et al. [99], respectively. Moreover, the remobilization of macro- and micronutrients under drought has been recently reported [100].

## 4. Volatile Emission by Phytopathogenic Organisms Represents a Great Opportunity to Improve Plant Tolerance towards Adverse Environmental Conditions

It has been repeatedly shown that beneficial microorganisms positively affect plant growth and development through the emission of volatile compounds [101]. Plants perceive biotic stimuli via a multitude of signaling compounds that originate from the interacting organisms. The nature of these compounds is diverse and includes carbohydrates, peptides, lipids, sterols, alcohols, aldehydes, and aromatic compounds [101,102]. Furthermore, Splivallo et al. [103] investigated the effect of fungal volatiles on the growth of *A. thaliana* in closed-chamber experiments and reported that volatiles released by truffles inhibited the development of *A. thaliana* and altered its oxidative metabolism, emphasizing a close interaction between the volatiles emitted by the fungi and the plant metabolism.

Ezquer et al. [104] demonstrated in a comprehensive study that volatile emissions from several microbial species, including pathogenic organisms, promoted the accumulation of remarkably high starch contents in the leaves of mono- and dicotyledonous plants. Moreover, using Arabidopsis mutants impaired in starch metabolism, the authors identified a complex transcriptionally and post-transcriptionally regulated network for microbial volatile-induced starch accumulation in which photoreceptors, starch synthase III, starch synthase IV, and NTRC (Nitrogen regulatory protein C) play a crucial role, associated with an increase of the reduced active form of the key starch biosynthetic enzyme ADPGlc pyrophosphorylase [105]. This pioneering discovery represented a technological breakthrough in the field of plant development and stress tolerance [104,105].

In a further study, Sánchez-López et al. [106] reported that the volatile compounds emitted by diverse phytopathogenic miroorganisms promote photosynthesis, plant growth, flowering, and starch accumulation, and that a highly conserved complex pathway is initiated upon Arabidopsis exposure to these volatiles, in which light processes and cytokinin-mediated signaling play an important function. The same authors demonstrated that volatiles emitted by the opportunistic fungal phytopathogen *Alternaria alternata* resulted in enhanced photosynthesis and accumulation of cytokinins and starch in the chloroplasts of a plastidic phosphoglucose isomerase (pPGI) mutant of Arabidopsis, leading them to the conclusion that the plant response to the volatiles bypasses the pPGI pathway through the transport of cytosolic hexoses and adenosine diphosphate-glucose into the chloroplast [107].

García-Gómez et al. [26] carried out a comprehensive investigation to evaluate the contribution and mode of action of volatile organic and inorganic compounds emitted by *A. alternata* and another fungal phytopathogen, *Penicillium aurantiogriseum*, on Arabidopsis plants. The authors demonstrated that the increased photosynthetic activity originating from the treatment with volatile compounds resulted in an increase of the metabolite glyceraldehyde phosphate, which enters the methylerythritol phosphate pathway to synthetize isoprenoid hormones. The latter initiate a cumulative change in the expression of genes involved in various metabolic processes [26]. Recently, García-Gómez et al. [108] provided evidence that the volatile compounds produced by *P. aurantiogriseum* modify root metabolism and architecture under both normal and drought conditions, resulting in improved nutrient and water use efficiency in Arabidopsis. In that study, a comprehensive metabolic, proteomic, and transcriptomic approach was undertaken to evaluate which regulatory networks were associated with the positive effects of the volatile compounds on root development. They clearly demonstrated that a complex process was initiated by the fungal volatiles that included changes in the expression of genes involved in the conversion of citrate to acetyl-CoA (ATP citrate synthase, ACLA-1), leading to enhanced levels of mevalonate-derived isoprenoids such as cytokinins that act as signaling compounds. They downregulated the expression of the iron-regulated transporter 1 (IRT1) and upregulated the expression of enzymes involved in the ethylene synthesis pathway. Furthermore, fungal volatile compounds downregulated the expression of invertases and aquaporin genes, cumulatively leading to a reduction of the turgor and consequently to root elongation [108]. The same authors found that Arabidopsis plants exposed to the volatiles emitted by *P. aurantiogriseum* displayed enhanced tolerance to drought stress [108], emphasizing the importance of these naturally produced compounds to the improvement plant growth under various environmental challenges.

**Figure 2:**
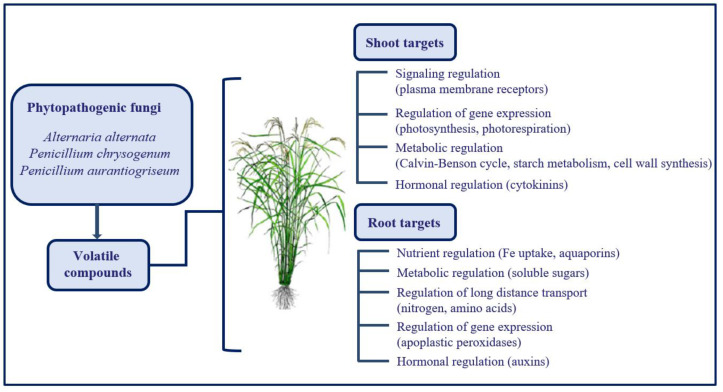
Schematic representation of key mechanisms targeted by volatiles of phytopathogenic fungi in normal and drought conditions. The scheme is based on observations reported by Sánchez-López et al. [106] and García-Gómez et al. [26,108]. The complex regulatory network initiated by these volatile compounds, which are emitted by phytopathogenic fungi, comprises regulation at the levels of gene expression, metabolites, nutrient allocation, and hormones, which together balance plant development and circumvent the possible growth loss caused by adverse environmental conditions.

Taken together, results published in recent years by Pozueta-Romero and coworkers [26,104,105,106,107,108] offer new and exciting biotechnological tools to improve plant development and tolerance in ambient and stress conditions through a source of environmentally friendly protective compounds. Moreover, this phenomenon increases our knowledge of the fundamental mechanisms in action during plant–microbe interactions. A summary of the events triggered in shoots and roots by the volatile compounds emitted by phytopathogenic organisms is presented in Figure 2.

## 5. Perspectives

In recent years, a large number of beneficial approaches have been proven and recommended to improve plant growth and tolerance towards rapidly changing environmental conditions. Several strategies rely on purposeful breeding, chemical treatments, and/or modification of stress-related genes. Beside these strategies, there is an urgent need to develop and utilize the existing plant protection resources that cover plant cognate genes from the ancestors of plants and/or environmentally friendly biostimulants originating from various organisms including bacteria, algae, or fungi. The recent advances reviewed in this article demonstrate that it is reasonable to expect successful outcomes for plant development and the resulting final yield that in turn secure food resources for the rapidly increasing global population of the future.

## Data Availability

Not applicable.
